# Reactive Oxygen Species Mediate Epstein-Barr Virus Reactivation by *N*-Methyl-*N’*-Nitro-*N*-Nitrosoguanidine

**DOI:** 10.1371/journal.pone.0084919

**Published:** 2013-12-20

**Authors:** Sheng-Yen Huang, Chih-Yeu Fang, Chung-Chun Wu, Ching-Hwa Tsai, Su-Fang Lin, Jen-Yang Chen

**Affiliations:** 1 Graduate Program of Biotechnology in Medicine of National Tsing Hua University and National Health Research Institutes, Hsinchu, Taiwan; 2 Institute of Biotechnology, Department of Life Sciences, National Tsing Hua University, Hsinchu, Taiwan; 3 Department of Microbiology, College of Medicine, National Taiwan University, Taipei, Taiwan; 4 National Institute of Cancer Research, National Health Research Institutes, Miaoli County, Taiwan; Karolinska Institutet, Sweden

## Abstract

N-nitroso compounds (NOCs) and Epstein-Barr virus (EBV) reactivation have been suggested to play a role in the development of nasopharyngeal carcinoma (NPC). Although chemicals have been shown to be a risk factor contributing to the carcinogenesis of NPC, the underlying mechanism is not fully understood. We demonstrated recently that N-methyl-N’-nitro-N-nitrosoguanidine (MNNG) enhances the genomic instability and tumorigenicity of NPC cells via induction of EBV reactivation. However, the mechanisms that trigger EBV reactivation from latency remain unclear. Here, we address the role of ROS in induction of EBV reactivation under MNNG treatment. EBV reactivation was induced in over 70% of EBV-positive NA cells and the promoter of Rta (Rp) was activated after MNNG treatment. Inhibitor experiments revealed ATM, p38 MAPK and JNK were activated by ROS and involved in MNNG-induced EBV reactivation. Significantly, ROS scavengers N-acetyl-L-cysteine (NAC), catalase and reduced glutathione inhibited EBV reactivation under MNNG and H_2_O_2_ treatment, suggesting ROS mediate EBV reactivation. The p53 was essential for EBV reactivation and the Rp activation by MNNG. Moreover, the p53 was phosphorylated, translocated into nucleus, and bound to Rp following ROS stimulation. The results suggest ROS play an important role in initiation of EBV reactivation by MNNG through a p53-dependent mechanism. Our findings demonstrate novel signaling mechanisms used by NOCs to induce EBV reactivation and provide a novel insight into NOCs link the EBV reactivation in the contribution to the development of NPC. Notably, this study indicates that antioxidants might be effective for inhibiting N-nitroso compound-induced EBV reactivation and therefore could be promising preventive and therapeutic agents for EBV reactivation-associated malignancies.

## Introduction

N-nitroso compounds (NOCs) have been classified by the International Agency for Research on Cancer as probably carcinogenic to humans (group 2A) [1]. NOCs are a group of compounds containing a nitroso group bound to a nitrogen atom. Humans are exposed to NOCs, not only through diet and cigarette smoking, but also through nitrogen-containing compounds which can be converted into nitroso derivatives in the gastrointestinal tract [2]. Epidemiological studies have associated human exposure to endogenous NOCs with several types of cancers including nasopharyngeal, esophageal, stomach, gastric, colorectal and bladder cancer [3,4]. 

Nasopharyngeal carcinoma (NPC) is a common head and neck cancer. The incidence rate is higher (25–30 per 100,000 person-years) in certain regions of southern China, Taiwan and Southeast Asia than others around the world (less than 1 per 100,000 person-years) [5,6]. Dietary, viral and genetic factors are implicated in the development of NPC [7]. Several studies have reported a close association between the consumption of salted fish and an excess risk of NPC in high-risk areas [8,9]. Volatile NOCs and their precursors are present in foods from NPC high risk areas and considered to be a potential etiological factor for NPC [10,11]. 

Epstein-Barr virus (EBV) infection has been associated with the development of many human malignancies, including NPC [12]. Retrospective studies revealed that NPC patients have elevated antibody titers to EBV antigens prior to diagnosis and prospective studies also showed that individuals with elevated antibodies against EBV have a higher risk of the development of NPC [13-15]. Moreover, seroepidemiological studies revealed that populations living in NPC high risk areas have high frequencies and serum titers of antibodies against EBV antigens [16,17]. Based on these observations, detection of antibodies against EBV antigens has been established as a standard test for NPC in high-risk populations [16,18,19]. Elevation of antibodies against EBV has been considered as a marker of EBV reactivation [18,20,21]. These antibody titers against EBV antigens are correlated with tumor burden, increase with the advancement of the stage of NPC [22,23], decrease after therapy with remission [22], and increase prior to relapse and metastasis [22,24]. These studies incriminate EBV reactivation as a cause of NPC. It is clear that EBV plays an etiological role in the carcinogenesis of NPC. However, infection with EBV is ubiquitous and persists latently in over 90% of the world’s population [25], but an extremely high incidence of NPC occur predominantly only in specific geographical regions [21]. Therefore, it is apparent that EBV infection alone is not a sufficient cause of NPC. Specific enviromental cofactors such as chemical exposure and dietary factors, which exist in high-incidence areas, may be critical for increasing the risk of NPC. Preserved food samples from NPC high risk areas were also found to contain inducers of EBV reactivation as well as NOCs [26]. Our recent study showed that N-methyl-N’-nitro-N-nitrosoguanidine (MNNG, a nitrosamide) could initiate EBV reactivation in EBV-positive NPC cells. Repeated treatment with a low dose of MNNG (0.1μg/ml) could induce EBV reactivation and had a synergistic effect with TPA/SB (inducers of EBV reactivation abundant in traditional Chinese herbal medicines and food sources, respectively) to enhance EBV reactivation [27]. Moreover, genome instability, invasiveness and the tumorigenicity of NPC cells were also enhanced after recurrent EBV reactivation [28]. These results strongly support the notion that chemical-induced EBV reactivation may contribute to the carcinogenesis of NPC [29]. However, the underlying mechanism by which N-nitroso compounds cause the initiation of EBV reactivation has not been extensively studied yet.

Over the past few decades, a considerable number of studies have demonstrated that cancer cells, compared to normal cells, are under high oxidative stress and this may alter metabolic activity significantly, stimulate cellular proliferation, and promote mutation and genomic instability [30]. Therefore, it is assumed that ROS are involved in the initiation, promotion and progression of tumors [31]. ROS are broadly defined as free radicals that contain unpaired electrons, such as superoxide (O_2_-) and hydroxyl radicals (·OH), and reactive non-radicals that are oxidizing agents or are easily converted into radicals, such as hydrogen peroxide (H_2_O_2_) [32]. There is evidence that viral infections (hepatitis C, HIV and influenza) are associated with an increased production of ROS and that could be involved in the pathogenesis [33,34]. EBV infection is also associated with the production of ROS [35,36] and this has been reported in EBV-associated diseases such as Burkitt’s lymphoma and NPC [37,38]. These studies imply that ROS are incriminated in EBV-associated disease. However, the effects of ROS on EBV infection remain unknown.

In this study, we use the ROS scavengers and donors to explore the role of ROS during EBV infection, in response to MNNG treatment. We find that ROS generation not only is required for efficient lytic reactivation by MNNG but also plays a crucial role in facilitating viral reactivation in response to the ROS donor, H_2_O_2_. Various signaling pathways including ATM, p38 MAPK and JNK, activated by ROS, are involved in MNNG induction of EBV reactivation and are responsible for multiple phosphorylation of p53. We also show that activation of the p53 protein is essential for MNNG induction of EBV reactivation and the mechanism of p53-mediated Rta promoter (Rp) activation requires p53 binding to Rp following ROS stimulation. These results suggest ROS play an important role in initiating EBV reactivation through p53-dependent mechanism. 

## Materials and Methods

### Cell lines

TW01 cells are EBV-negative NPC cell line derived from nasopharyngeal tumors from Taiwanese patients [39]. EBV-positive cell lines, NA, HA and H1299A cells respectively derived from TW01, HONE-1 (NPC cell lines derived from a Chinese NPC patient) and H1299 cells (human lung adenocarcinoma cells with a deletion of the p53 gene), were established by infection with a neomycin-resistant recombinant Akata-EBV and selected by G418 [40]. All cells were cultured in Dulbecco's modified Eagle medium supplemented with 10% fetal bovine serum (HyClone, Waltham, MA) at 37°C with 5% CO_2_. G418 (400 μg/ml, Ameresco, Solon, OH) was added to the medium of NA, HA and H1299A cells to maintain the EBV genome. C666-1, which consistently carries the EBV in long-term cultures, is a subclone of its parental cell line C666, derived from a southern Chinese NPC patient [41]. The cells were cultured in RPMI-1640 medium (HyClone, Waltham, MA) supplemented with 5% fetal bovine serum (HyClone, Waltham, MA) at 37°C with 5% CO2.

### Plasmids

Plasmids expressing siRNA targeting p53 and green fluorescence protein (GFP) were constructed by cloning siRNA sequences into pSuper vectors (Oligoengine) described in a previous publication [42]. Plasmids expressing wild-type p53 were kindly provided by Dr. Sheau-Yann Shieh (Institute of Biomedical Sciences, Academia Sinica, Taipei, Taiwan). The reporter plasmids driven by the BRLF1 (Rp, nucleotides nt. 106177 to 107144) or the BZLF1 promoter sequence (Zp, nucleotides nt. 103182 to 103415) of the EBV genome from B95-8 were amplified by PCR and ligated into the pGL2-basic vector (Promega). 

### Antibodies and chemicals

Antibodies against Zta [43] and EA-D [44] were generated in the laboratories. Anti-Rta antibody was obtained from Argene. Anti-phospho-ATM (1981), anti-phospho-ERK1/2 (Thr202/Tyr201), anti-phospho-SAPK/JNK (Thr183/Tyr182), anti-phospho-p38 MAPK (Thr180/Tyr182), Anti-ATM, anti-ERK1/2, anti-SAPK/JNK, anti-p38 MAPK, anti-β-actin antibody and phospho-p53 antibody Sampler Kit were purchased from Cell Signaling Technology. FITC-conjugated anti-mouse antibody was purchased from Upstate. Anti-Sp1 and anti-p53 were obtained from Santa Cruz. *N*-methyl-N’-nitro-*N*-nitrosoguanidine (MNNG), 12-O-tetradecanoylphorbol-1, 3-acetate (TPA), dihydroethidium (DHE), N-acetyl-L-cysteine (NAC), catalase, reduced glutathione, H_2_O_2_, caffeine, U0126, SB203580, SP600125, rottlerin, wortmannin, Bay11-7082 were purchased from Sigma-Aldrich.

### Western Blotting

Cells were lysed in lysis buffer containing 3.3% SDS, 1.67 M urea and 4.4% 2-mercaptoethanol. The BCA protein assay kit (Pierce, USA) was used to determine protein concentrations using bovine serum albumin as a standard. Cellular lysates were loaded onto 10% SDS-polyacrylamide gels. The protein bands were then electrophoretically transferred to Hybond-C membranes (Amersham). Membranes were probed with appropriate amounts of primary antibody and followed with a horseradish peroxidase-conjugated secondary antibody. Antibody reactions were detected using the ECL Western blotting detection reagent (Amersham) according to the manufacturer's recommendations.

### Immunofluorescence assay

Cells were washed with phosphate buffered saline (PBS) followed by fixation and premeabilization by exposure to ice-cold 100% methanol for 15 min. The cells were submitted to immunofluorescence staining using antibodies against the Zta, EA-D or p53 protein as primary antibodies, and then FITC-labeled goat anti-mouse IgG as the secondary antibody. The nuclei were stained with Hoechst 33258 (1μg/ml) for 1 min and washed with PBS, and the coverslips were mounted on slides and images were captured by fluorescence microscope.

### Quantitative reverse transcription-PCR (qRT-PCR)

Total RNA was extracted by using Trizol reagent (Invitrogen). Reverse transcription of 1 μg RNA was performed in a 20 μl SuperScript III reaction mixtures (Invitrogen) according to the manufacturer’s instructions. One tenth of the resulting cDNAs were used for each qPCR composed of 4 μl diluted cDNA, 5 μl Power SYBR Green Master Mix (Applied Biosystems) and 1 μl primer mix (2 μM). Three independent experiments were performed and each individual samples were performed in triplicate. The amount of RNA present in each sample was normalized to 18S rRNA. The primers used in the present study were as follows: Zta-forward (5’-GAGTC AACAT CCAGG CTTGG-3’) and Zta-reverse (5’-GCAGC ACTAC CGTGA GGTG-3’); Rta forward (5’-TGGTC AGTTC GTCCA AATGG-3’) and Rta-reverse (5’-CCAGA AGGAG GAAGC AGCC-3’); 18S rRNA-forward (5’-CGCCG CTAGA GGTGA AATTC-3’) and 18S rRNA-reverse (5’-TTGGC AAATG CTTTC GCTC-3’). The reaction was performed on StepOnePlus Real-Time PCR system (Applied Biosystems).

### Measurement of ROS

Cells were treated with MNNG for 24 h, and finally co-incubated with 10 µM ROS-sensitive probe DHE for 1 h. After incubation, cells were harvested by trypsinization. The labeled samples were analyzed by the FACScan flowcytometer and the CellQuest software (BD Biosciences, San Jose, CA). Ten thousand events were collected from each sample. The intracellular levels of ROS were calculated as the mean fluorescence intensity (MFI).

### Promoter activity assay

Two μg Rp or Zp-firefly luciferase reporter plasmid and 0.1 μg renilla luciferase reporter plasmid (pWP1, Promega) as a control were co-transfected into TW01 cells. Twenty-four hours post-transfection, the cells were treated with MNNG for another 24 h and then harvested and subjected to the luciferase assay using a Dual-Glo assay kit (Promega). Luciferase activity was measured for 10s with a Lumat LB9501 luminometer (Berthold Systems, Inc.). The firefly luciferase activity of each sample was normalized to the renilla luciferase activity. The fold of relative promoter activity was calculated by dividing that of the drug-treated transfectants by that of solvent control transfectants. 

### Chromatin-Immunoprecipitation (ChIP) Assay

Cells were trypsinized and cross-linked with formaldehyde for 10 min. The cross-linking was stopped by adding glycine for 10 min. Cell pellets were resuspended in cell lysis buffer (50 mM HEPES-KOH pH7.5, 140mM NaCl, 1 mM EDTA, 10% glycerol, 0.5% NP40, 0.25% triton X-100). Nuclei were pelleted and resuspended in nuclei lysis buffer (50 mM Tris pH 8.1, 10 mM EDTA, 1% SDS) containing phosphatase inhibitor and complete protease inhibitor cocktail (Roche, Nutley, NJ). Subsequently, DNA-bound protein lysates were sonicated to yield 500–1,000 bp DNA fragments and incubated in antibody-containing ChIP dilution buffer (0.01% SDS, 1.1% TritonX-100, 1.2 mM EDTA, 16.7 mM Tris–HCl pH 8.1, 167 mM NaCl, 1 mM DTT) at 4°C overnight on a rotating rocker. The immunocomplexes were precipitated using 200 μl of protein G-Sepharose beads (GE Healthcare, Waukesha, WI) at 4°C for 2 h. After sequential washes with low salt buffer (20 mM, Tris pH 8.0, 2 mM EDTA, 150 mM NaCl, 1% Triton, 0.1% SDS), high salt buffer (20 mM Tris pH 8.0, 2 mM EDTA, 500 mM NaCl, 1% Triton, 0.1% SDS), LiCl buffer (10 mM Tris pH 8.1, 0.25 M LiCl, 1 mM EDTA, 1% NP40, 1% IGEPAL) and TE buffer, the DNA-bound immunocomplexes were eluted and the DNA were extracted by PCR clean up kit. PCR reaction which specifically amplified the -442 to -2 region of Rp was performed using forward primer 5’-TGTGT GAGGT CTCAC CTGGA-3’ and reverse primer 5’-AGTAA TCCAT GTCAG CCGGC-3’. The amplification of -221 to +12 of Zp was performed using forward primer 5’-GCAAG GTGCA ATGTT TAGTG AG-3’ and reverse primer 5’- CCATG CATAT TTCAA CTGGG C-3’.

## Results

### MNNG induces EBV reactivation in EBV-positive NPC cells

**Figure 1:**
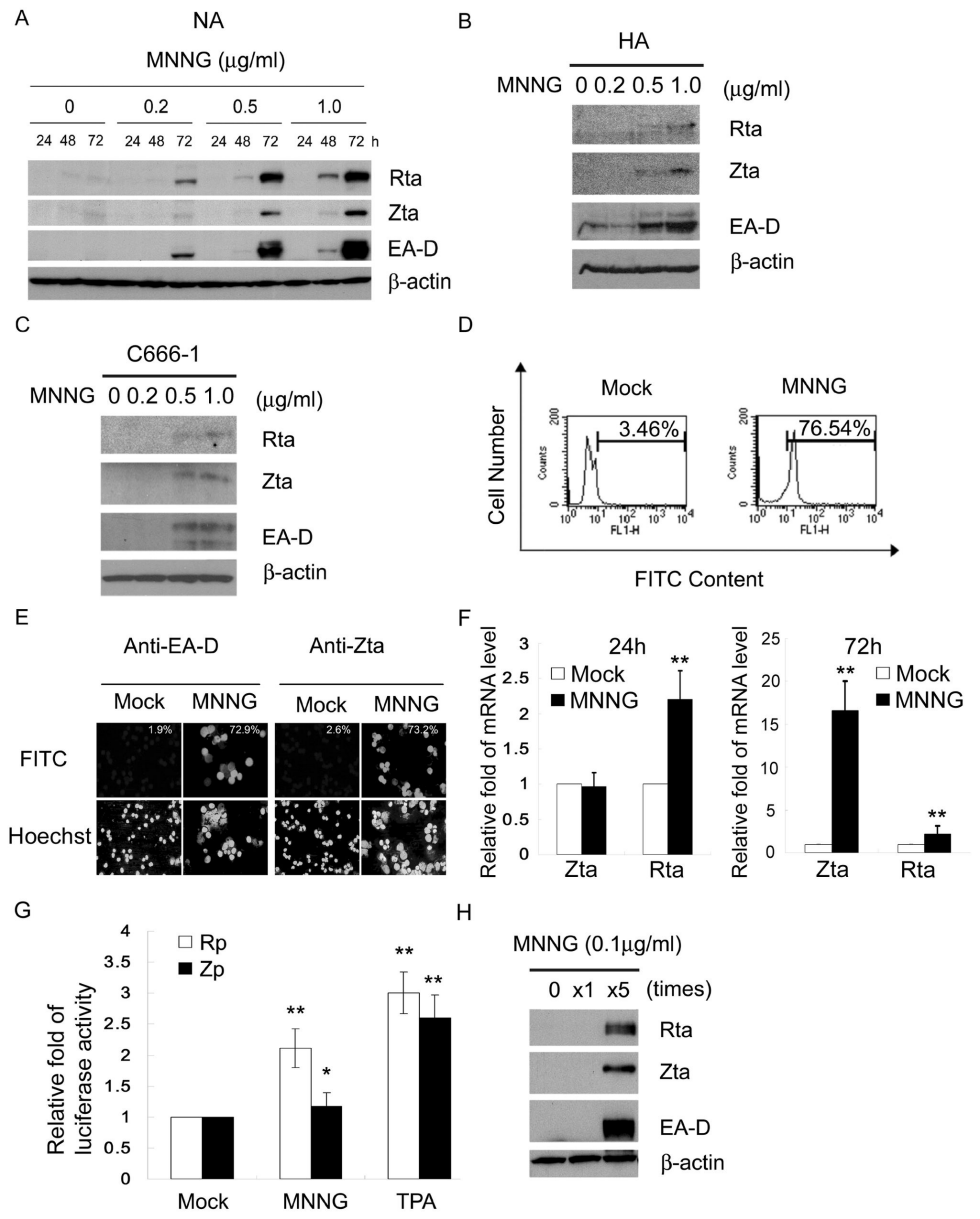
MNNG induces EBV reactivation in EBV-positive NPC cells. (A) MNNG induced EBV entry to the lytic cycle in a dose-dependent manner. NA cells were mock treated or treated with various concentrations of MNNG. At 24, 48 and 72 h post-treatment the cells were harvested and subjected to immunoblot analysis of the expression of the lytic EBV proteins, Rta, Zta, EA-D and cellular β-actin as an internal control. (B) MNNG induced EBV entry to the lytic cycle in HA cells and (C) C666-1 cells. The cells were mock treated or treated with various concentrations of MNNG. At 72 h post-treatment the cells were harvested and subjected to immunoblot analysis. (D) Seventy six percent of NA cells were induced into EBV reactivation after MNNG treatment. NA cells were mock treated or treated with MNNG (1μg/ml) for 72 h. Flow cytometry was performed to detect EA-D expressing NA cells. Numbers indicates the percentages of EA-D-presenting cells. Each assay was performed with 10,000 cells. (E) Over 70% of NA cells were induced into EBV reactivation after MNNG treatment. NA cells were mock treated or treated with MNNG (1μg/ml) for 72 h. The cells were first stained with anti-EBV EA-D or Zta antibodies and then FITC-conjugated second antibody. The locations of cell nuclei in the same fields were revealed by staining with Hoechst 33258. Numbers indicates the percentages of EA-D- or Zta-presenting cells. (F) MNNG-mediated Zta and Rta transcriptional activations were validated by quantitative RT-PCR. RNAs were extracted from NA cells treated with MNNG for 24h/72h or mock treated. (G) MNNG increased the activity of Rta promoter (Rp). Reporter constructs driven by the Rp or Zp were used in this luciferase reporter assay. TW01 cells were transfected with reporter plasmids of Rp or Zp and co-transfected with pWP1 as a control. After 24 h post-transfection the cells were mock treated or treated with MNNG (1μg/ml) for another 24 h. Cell lysates were harvested for luciferase activity assay (H) Repeating treatments with low-dose MNNG induced EBV reactivation. NA cells were repeatedly treated with MNNG (0.1 μg/ml) once daily for 5 days. Cell lysates were harvested and subjected to immunoblotting. *: *p*<0.05, **: *p*<0.01, compared to mock treatment of the same mRNA level or promoter.

To examine the effect of MNNG on the induction of EBV reactivation, EBV-positive NA cells were treated with MNNG at various concentrations for 24-72 h. The cell lysates were subjected to immunoblotting and viral reactivation was assayed by the detection of EBV immediate early proteins, BRLF1 (Rta) and BZLF1 (Zta), and the early antigen, BMRF1 (EA-D). As shown in Figure 1A, treatment with 0.2 μg/ml MNNG did not lead to marked viral reactivation in NA cells. However, the level of viral reactivation increased with increasing concentration of MNNG from 0.5 to 1 μg/ml. Similar effect was observed in EBV-positive HA (Figure 1B) and C666-1 cells (Figure 1C). Under the condition of 1 μg/ml for 72 h, more than 70% NA cells were induced into the EBV lytic cycle, as determined by flow cytometry using EAD-staining (Figure 1D) or immunofluorescence assays for EAD/Zta (Figure 1E). Since the immediate early proteins, Rta and Zta, play key roles in initiating EBV reactivation [45], we performed quantitative mRNA analysis to examine the mRNA level of Rta and Zta. Figure 1F shows the amount of Rta mRNA was increased to about 2.2-fold by MNNG treatment (1 μg/ml) for 24h, which is higher than the solvent control, while the amount of Zta mRNA was not significantly increased. However, the amount of Zta mRNA was markedly increased by MNNG treatment (1 μg/ml) for 72h to about 16.6-fold higher than the solvent control. The results suggest that MNNG initiates EBV reactivation may through induction of Rp activation and the expression of Rta subsequently leads to activation of Zta expression implying that MNNG induces EBV reactivation may mainly through induction of Rp activation. To further explore whether MNNG induces EBV reactivation through induction of the promoters of these proteins, the reporter plasmid of Rta or Zta promoter (Rp or Zp) was transfected separately into the EBV-negative TW01 cells. As shown in Figure 1G, the Rp was significantly activated by MNNG (1 μg/ml for 24 h) to levels about 2.1-fold, but the Zp was only slightly activated to 1.2-fold higher than the solvent control. This result indicates that MNNG induction of EBV reactivation may be mainly through increasing the activities of Rp. Residents in NPC high risk areas maybe exposed to nontoxic doses of NOCs for a long time before the development of NPC. Considering this physiological relevance, repeated treatment with a low dose and non-cytotoxic concentration of MNNG (0.1μg/ml), once daily for 5 days, was investigated in NA cells. The result showed that repeated treatment with non-cytotoxic MNNG significantly induced viral reactivation (Figure 1H), implying that exposure to NOCs for a long time may increase the risk of NPC development via the reactivation of EBV. 

### ROS, ATM, p38 MAPK and JNK signaling pathways are involving in MNNG-induced EBV reactivation

**Figure 2:**
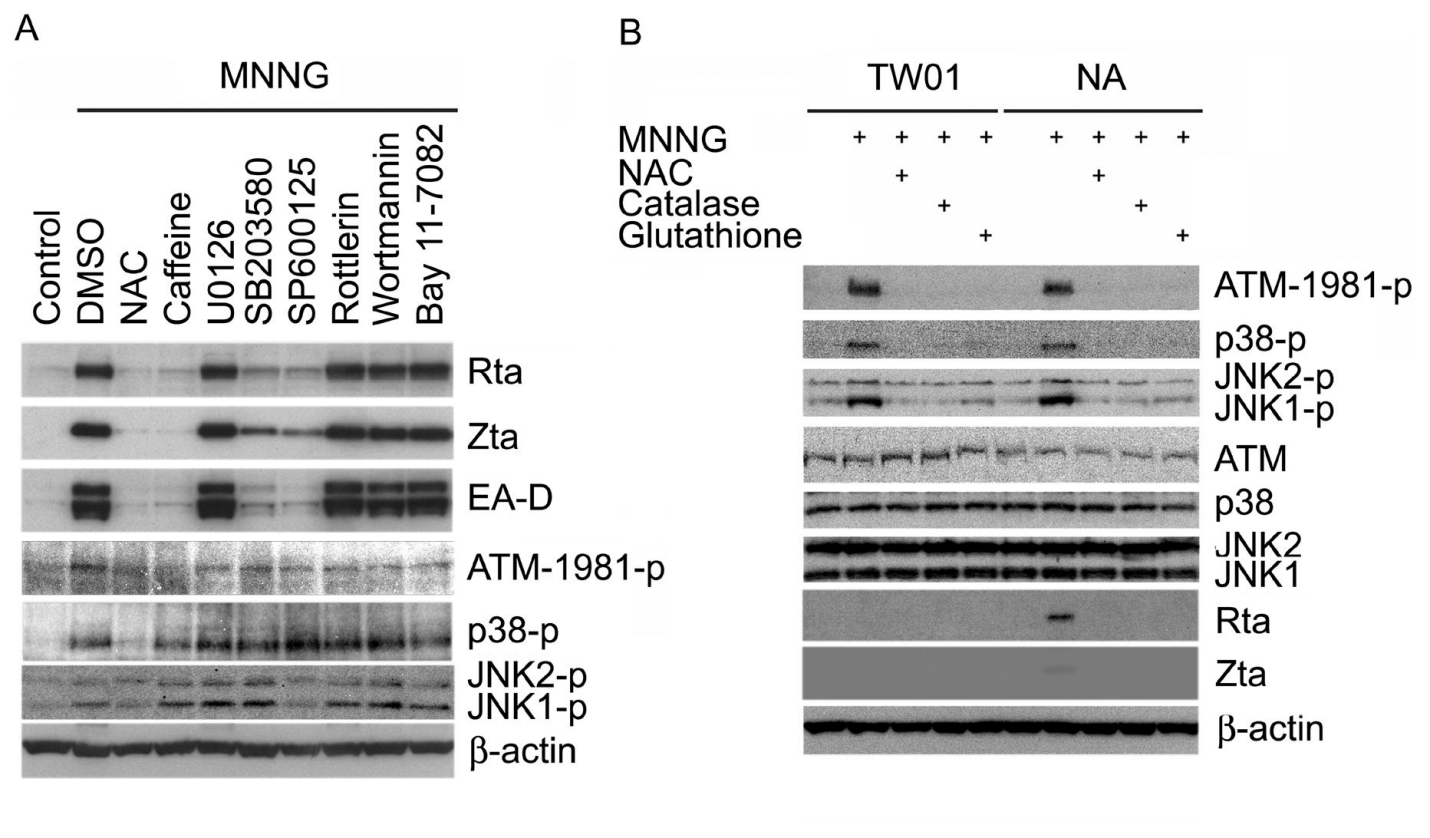
ROS, ATM, p38 MAPK and JNK pathways are involved in MNNG-induced EBV reactivation. (A) Examination of signaling pathways involved in MNNG-induced EBV reactivation. NA cells were pretreated with DMSO control or inhibitors including NAC (1mM), caffeine (1mM), SB203580 (10μM), SP600125 (10μM), U0126 (10μM), rottlerin (2.5μM), wortmannin (100nM) or Bay 11-7082 (5μM) for 1 h and then treated with MNNG (1μg/ml) for additional 72 h. Lytic EBV proteins, Rta, Zta and EA-D, and phosphorylated forms of ATM, p38 MAPK and JNK1/2 were detected by immunoblotting. Cellular β-actin was as an internal control. (B) MNNG activated phosphorylation of ATM, p38 MAPK and JNK through ROS generation. TW01 and NA cells were pretreated with ROS inhibitors, including NAC (1mM), catalase (1000 unit/ml) and reduced glutathione (1mM) for 1 h, and then treated with MNNG (1μg/ml) for another 24 h. Total forms and phosphorylated forms of ATM, ERK1/2, p38 MAPK and JNK1/2, and lytic EBV proteins, Rta and Zta were detected by immunoblotting. Cellular β-actin was as an internal control.

Previous studies have shown that MNNG could rapidly induce ROS production [46] and activate ATM and MAPK signaling pathways [47]. Many chemicals which activate the EBV reactivation induce a variety of signal transduction pathways, including MAPKs, ATM, protein kinase C, and NF-κB, and these kinases also have been shown to be involved in the induction of lytic EBV transcription following various stimuli [48,49]. To determine which pathways were involved in MNNG-induced EBV reactivation, kinase inhibitors, including NAC (ROS scavenger), caffeine (ATM inhibitor), U0126 (ERK inhibitor), SB203580 (p38 MAPK inhibitor), SP600125 (JNK inhibitor), rottlerin (PKC inhibitor), wortmannin (PI3K inhibitor) or Bay 11-7082 (NFκB inhibitor) were utilized. Pretreatment of NA cells with NAC (1mM), caffeine (1mM), SB203580 (10μM) or SP600125 (10μM), but not U0126 (10μM), rottlerin (2.5μM), wortmannin (100nM) or Bay 11-7082 (5μM), blocked MNNG-induced EBV reactivation (Figure 2A). The phosphorylation of ATM, p38 MAPK and JNK1/2 were inhibited by NAC or respective inhibitors. However, SB203580 inhibits p38 MAPK catalytic activity by binding to the ATP-binding pocket, but does not inhibit the phosphorylation of p38 MAPK. This result indicates that ROS, ATM, p38 MAPK and JNK are involved in MNNG-induced EBV reactivation. Moreover, it has been shown that ROS can activate the signaling pathways of ATM, p38 MAPK or JNK. To test whether ROS play a key role in inducing these signaling pathways, ROS scavengers were used in the study. Figure 1B shows that MNNG (1μg/ml) can strongly induce ATM, p38 MAPK and JNK phosphorylation in TW01 and NA cells. MNNG do induce the phosphorylation of these kinases in EBV-negative TW01 cells, which do not contain EBV genome, that implying lytic products of EBV were not responsible for this phenomenon. Moreover, activation of these kinases by MNNG was completely abolished after pretreatment with NAC (1mM), catalase (1000 unit/ml) or reduced glutathione (1mM). These results indicate ROS is the upstream effecter of MNNG-induced phosphorylation of ATM, p38 MAPK and JNK, and MNNG induces EBV reactivation by activating these kinases.

### MNNG induces EBV reactivation through ROS generation

**Figure 3:**
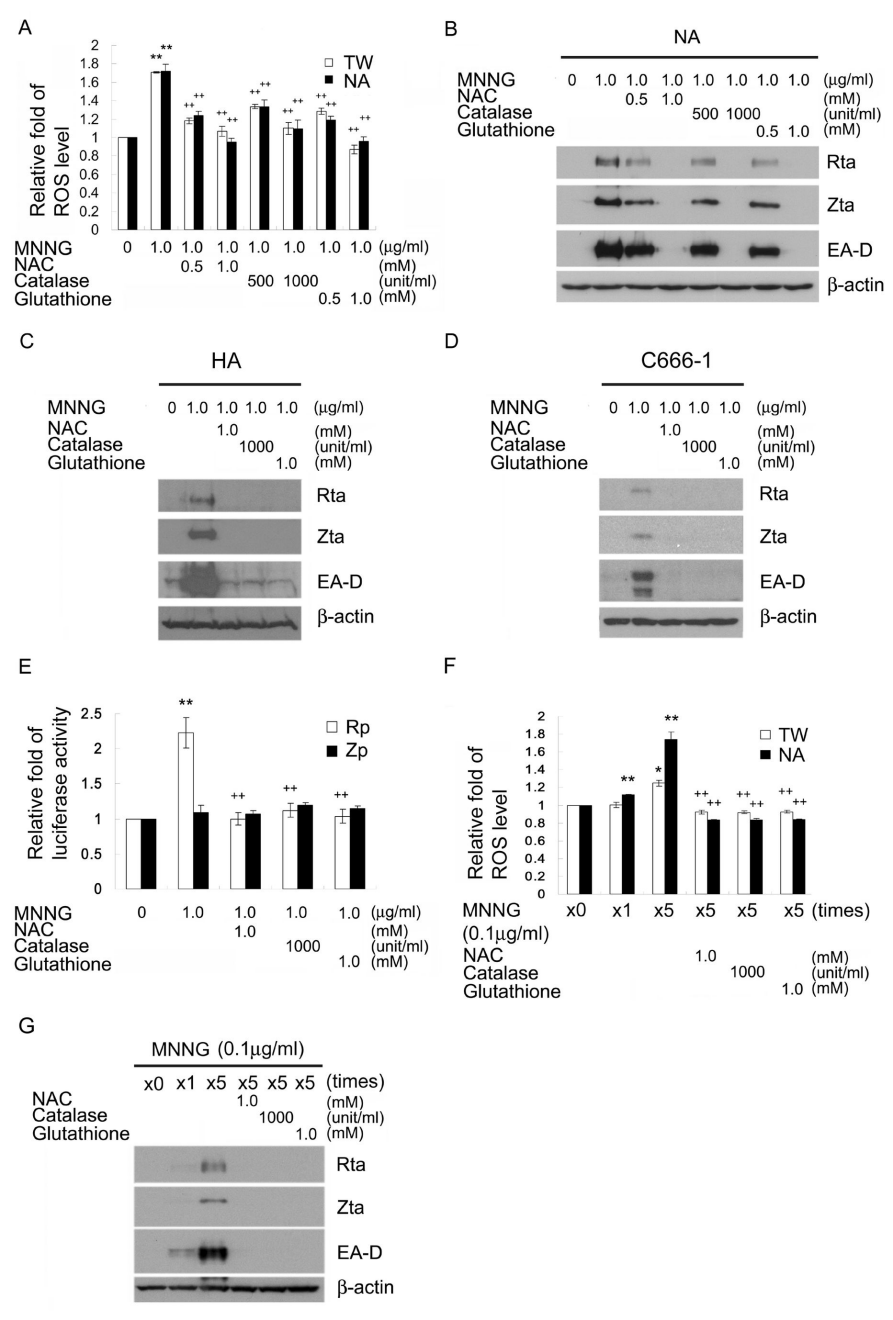
ROS mediates EBV reactivation by MNNG. (A) MNNG increased the intracellular ROS levels in TW01 or NA cells and the ROS scavengers reduced MNNG-induced ROS production. Cells were pretreated with ROS scavengers including NAC (0.5 or 1mM), catalase (500 or 1000 unit/ml) and reduced glutathione (0.5 or 1mM) for 1 h and then mock treated or treated with MNNG (1μg/ml) for 24 h. Cells were stained with the ROS-sensitive probe dihydroethidium (DHE) for 1 h and the fluorescence levels were measured by flow cytometry. (B) ROS scavengers reduced the expression of Rta, Zta and EA-D proteins induced by MNNG in NA, (C) HA and (D) C666-1 cells. After pretreatment with ROS scavengers for 1 h and MNNG (1μg/ml) treatment for another 72 h, cells were harvested and subjected to immunoblotting using antibodies against the lytic EBV proteins, Rta, Zta, EA-D and cellular β-actin as an internal control. (E) The activity of Rta promoter was induced by MNNG and the effect was inhibited by ROS scavengers. Luciferase activities were measured in TW01 cells transfected with the reporter plasmids of Rp or Zp for 24 h. The cells were pretreatment with ROS scavengers for 1 h, following by treating with MNNG (1μg/ml) for additional 24 h and harvested for luciferase activity assay. (F) Repeated treatment with a low dose concentration of MNNG increased the ROS level. TW01 and NA cells were repeatly treated with MNNG (0.1μg/ml) once a day for 5 days and pretreated with NAC (1mM), catalase (1000 unit/ml) or reduced glutathione (1mM) for 1 h on each occasion. Cells were then stained with the ROS-sensitive probe dihydroethidium (DHE) for 1 h and their fluorescence levels were measured by flow cytometry. (G) ROS scavengers inhibited EBV reactivation by repeated treatment with a low dose MNNG (0.1μg/ml). The treatment procedure was the same as described in (F). Cells were harvested and subjected to immunoblotting. *: *p*<0.05, **: *p*<0.01, compared to mock treatment of the same cell line or the same promoter; +: *p*<0.05, ++: *p*<0.01, compared to MNNG treatment or MNNG treatment 5 times of the same cell line or the same promoter.

To confirm that MNNG induces ROS generation, we used the fluorescent dye dihydroethidium (DHE) to determine the intracellular level of ROS after MNNG treatment. MNNG (1μg/ml) not only increased the ROS level to 1.7-fold in TW01 and NA cells (Figure 3A) but also induced EBV reactivation in NA cells (Figure 3B). To determine whether MNNG-induced ROS is required for EBV reactivation, we used ROS scavengers to reduce the intracellular ROS levels. As shown in Figure 3A, pretreatment with NAC (1mM), catalase (1000 unit/ml) and reduced glutathione (1mM) inhibited MNNG induction of ROS generation, respectively, by 1.1-fold, 1.1-fold and 0.9-fold in TW01 cells and 1.0-fold, 1.1-fold and 1.0-fold in NA cells. As expected, ROS scavengers inhibited MNNG-induced EBV reactivation in a dose-dependent manner (Figure 3B). Similarly, ROS scavengers inhibited MNNG-induced EBV reactivation were also observed in EBV-positive HA (Figure 3C) and C666-1 cells (Figure 3D). Consistent with these results, Rp activity was induced 2.2-fold by MNNG and the induction effect was inhibited by ROS scavengers (Figure 3E). In addition, 5 times repeated treatment with a low dose and non-cytotoxic concentration of MNNG (0.1μg/ml) increased the ROS level to 1.3-fold in TW01 and 1.7-fold in NA cells (Figure 3F). ROS scavengers also effectively blocked induction of ROS and inhibited EBV reactivation by repeated treatment with a low dose MNNG (0.1μg/ml) (Figure 3F, G). These results indicate that ROS are required for MNNG-induced EBV reactivation, and antioxidants can effectively inhibit EBV entering lytic replication following induction by MNNG. 

### H_2_O_2_ induces EBV reactivation

**Figure 4:**
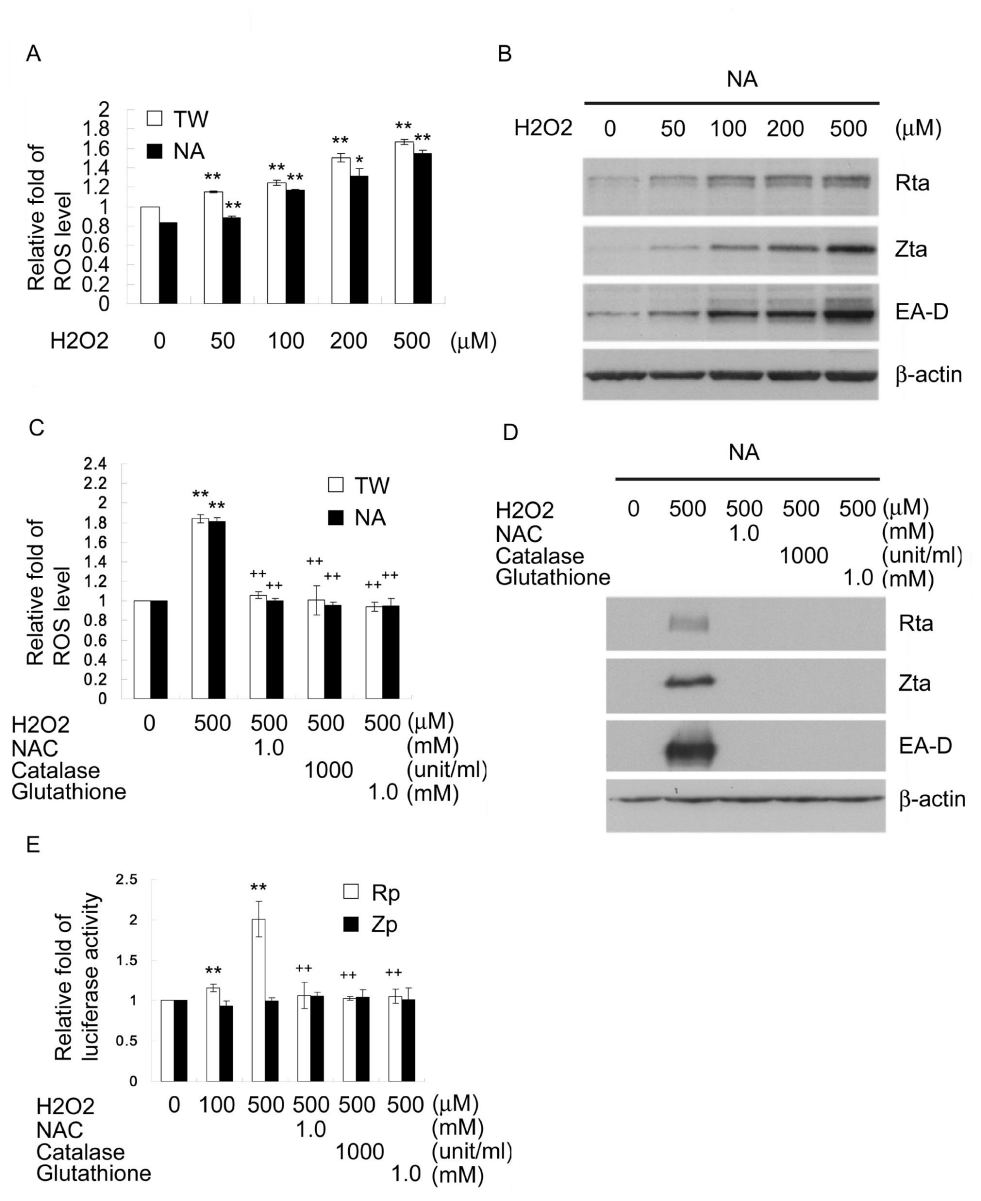
H_2_O_2_ induces EBV reactivation. (A) H_2_O_2_ increased intracellular ROS levels in a dose-dependent manner. TW01 and NA cells were treated with various concentrations of H_2_O_2_ for 24 h. Cells were then stained with the ROS-sensitive probe dihydroethidium (DHE) for 1 h and their fluorescence levels were measured by flow cytometry. (B) H_2_O_2_ induced EBV reactivation in a dose-dependent manner. NA cells were treated with various concentrations of H_2_O_2_ for 72 h. Cell lysates were harvested and subjected to immunoblotting analysis for detection of the expression of the EBV lytic proteins, including Rta, Zta, EA-D, and cellular β-actin was used as an internal control. (C) ROS scavengers reduced H_2_O_2_-induced ROS production. TW01 and NA cells were pretreated with NAC (1mM), catalase (1000 unit/ml) or reduced glutathione (1mM) for 1 h and then treated with MNNG (1μg/ml) for 24 h. Cells were then stained with the ROS-sensitive probe dihydroethidium (DHE) for 1 h and their fluorescence levels were measured by flow cytometry. (D) ROS scavengers inhibited H_2_O_2_-induced EBV reactivation. NA cells were pretreated with NAC (1mM), catalase (1000 unit/ml) or reduced glutathione (1mM) for 1 h and then treated with MNNG (1μg/ml) for 72 h. Cell lysates were harvested and subjected to immunoblotting for detection of the expression of the lytic EBV proteins. (E) The activity of Rta promoter was induced by H_2_O_2_, and the induction effect was inhibited by ROS scavengers. Luciferase activities were measured in TW01 cells transfected with the reporter plasmids of Rp or Zp. The cells were pretreatment with ROS scavengers for 1 h, followed by treating with H_2_O_2_ (500 μM) for another 24 h, and harvested for luciferase activity assay. *: *p*<0.05, **: *p*<0.01, compared to mock treatment of the same cell line or on the same promoter; +: *p*<0.05, ++: *p*<0.01, compared to H_2_O_2_ (500 μM) treatment of the same cell line or the same promoter.

To confirm the effect of ROS on lytic EBV replication, we sought to determine whether an increase in intracellular ROS levels is sufficient to induce EBV reactivation. As shown in Figure 4A, a dose-dependent induction of ROS was observed following H_2_O_2_ treatment of TW01 and NA cells. Furthermore, H_2_O_2_ also consistently induced EBV reactivation in a dose-dependent manner in NA cells (Figure 4B). H_2_O_2_ (500μM) increased intracellular ROS levels to 1.8-fold in TW01 and NA cells (Figure 4C), while NAC (1mM), catalase (1000 unit/ml) and reduced glutathione (1mM) effectively abolished production of ROS and inhibited EBV reactivation (Figure 4C, D). Rp activity was induced 2.0-fold by H_2_O_2_ (500μM) and the induction effect was inhibited by ROS scavengers (Figure 4E). These results suggest that an increase in intracellular ROS levels can be the trigger that reactivates EBV from latency.

### p53 is required for MNNG-induced EBV reactivation

**Figure 5:**
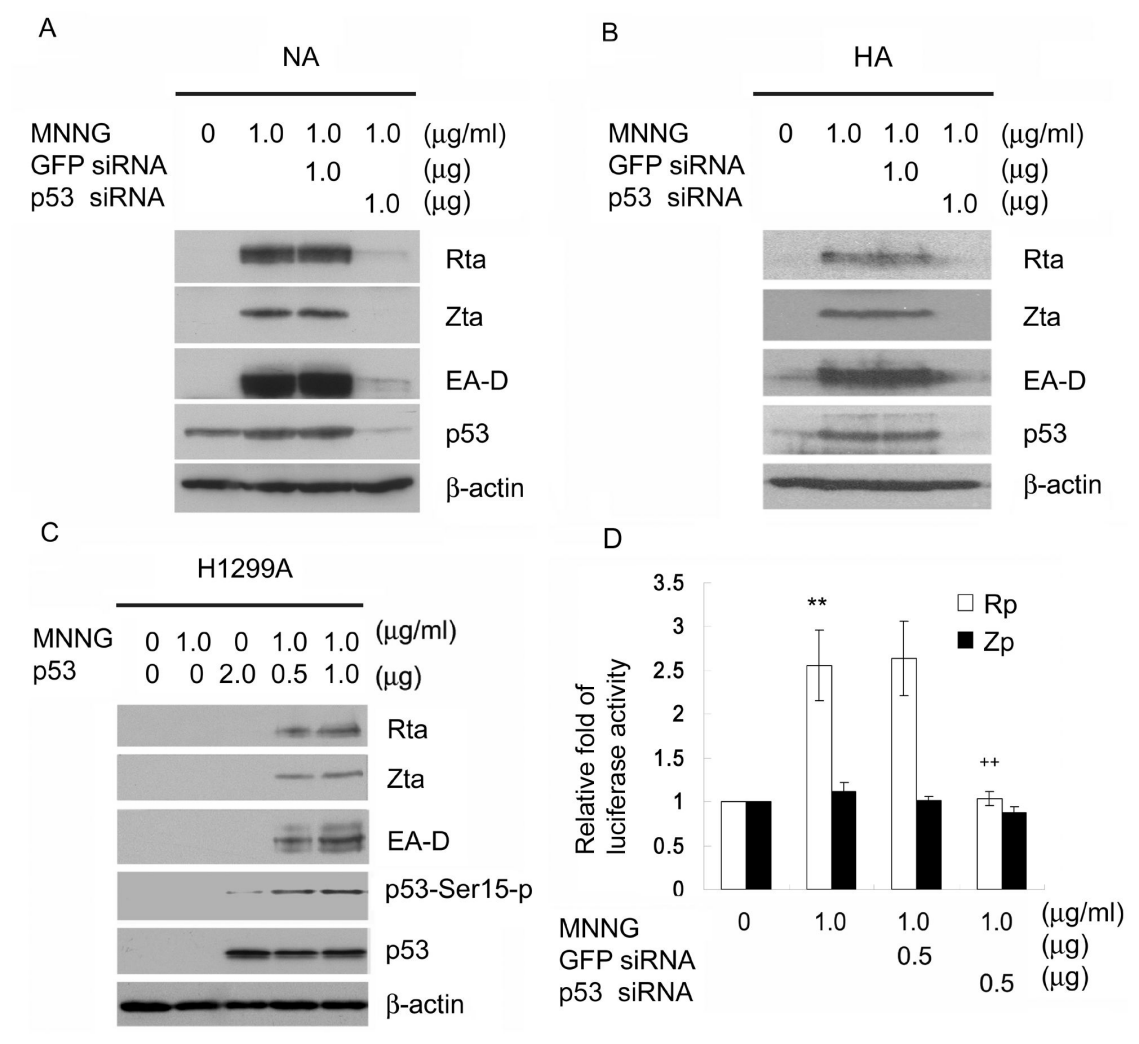
p53 is required for MNNG-induced EBV reactivation. (A) p53 is required for MNNG-induced lytic immediate early and early protein expression in NA and (B) HA cells. NA and HA cells were knocked down for p53 expression using p53 siRNA or control GFP siRNA. The cells were treated with MNNG (1μg/ml) for 72 h, and immunoblot analysis was performed to detect lytic protein expression. (C) Restoration of p53 effectively induced EBV reactivation after MNNG treatment. p53-null H1299A cells were transiently transfected with a p53-expression plasmid for 24 h, followed by MNNG (1μg/ml) treatment for an additional 72 h. Cell lysates were harvested and subjected to immunoblotting for detection of the expression of the EBV lytic proteins. (D) p53 is essential for the activation of Rta promoter activity by MNNG. TW01 cells were co-transfected with the Rp/Zp reporters and p53 siRNA or GFP siRNA. The transfected cells were then treated with MNNG (1μg/ml) for 24 h and harvested for luciferase activity assay. *: *p*<0.05, **: *p*<0.01, compared to mock treatment of the same promoter; +: *p*<0.05, ++: *p*<0.01, compared to MNNG treatment of the same promoter.

ROS are known to activate p53 [50] and it has been reported that p53 participates in chemical-induced EBV reactivation [51,52]. We used a small interfering RNA (siRNA) expression approach to determine whether MNNG-induced EBV reactivation requires p53. NA cells were knocked down for p53 expression using p53 siRNA or GFP siRNA as a control, and then treated with MNNG (1μg/ml). As shown in Figure 5A, MNNG increased p53 protein expression and induced EBV reactivation. However, the induction of EBV reactivation was highly attenuated in cells knocked down for p53 expression, but not in cells knocked down for GFP expression (Figure 5A). In order to further confirm the necessity of p53 for MNNG mediated EBV reactivation is not a cell line dependent event, HA cells were examined by p53 siRNA knockdown in MNNG mediated EBV reactivation. The similar effect was observed in HA cells (Figure 5B) as NA cells, implying that p53 is essential for MNNG mediated EBV reactivation. To verify the requirement of p53 for MNNG-induced EBV reactivation, a plasmid expressing wild-type p53 was introduced into EBV-positive but p53-null H1299A cells. Without p53 expression, MNNG loses its ability to induce EBV reactivation in H1299A cells. Notably, restoration of p53 expression effectively induced EBV reactivation by MNNG treatment in a dose-dependent manner (Figure 5C). These data suggest that p53 expression is critical for MNNG-induced EBV reactivation. Moreover, the failure of p53 restoration without MNNG treatment to induce reactivation indicated the importance of post-translational modification of the p53 protein by MNNG during MNNG-induced EBV reactivation. Taken together, these data suggest that the ability of MNNG to induce EBV reactivation is p53-dependent and the post-translational modification of p53 may be required for activation of EBV by MNNG. In addition, Rp and Zp reporter assays were performed to test whether p53 is involved in regulating the initial step of EBV lytic entry by MNNG. Rp or Zp was activated by MNNG (1 μg/ml for 24 h) at levels about 2.6-fold or 1.1-fold higher than the solvent control, but was reduced to 1.0-fold or 0.9-fold when the expression of p53 was abrogated by p53 siRNA in TW01 cells (Figure 5D). These results suggest that the ability of MNNG to activate the Rp requires the presence of p53. 

### p53 is multiply phosphorylated by ATM, p38 MAPK and JNK

**Figure 6:**
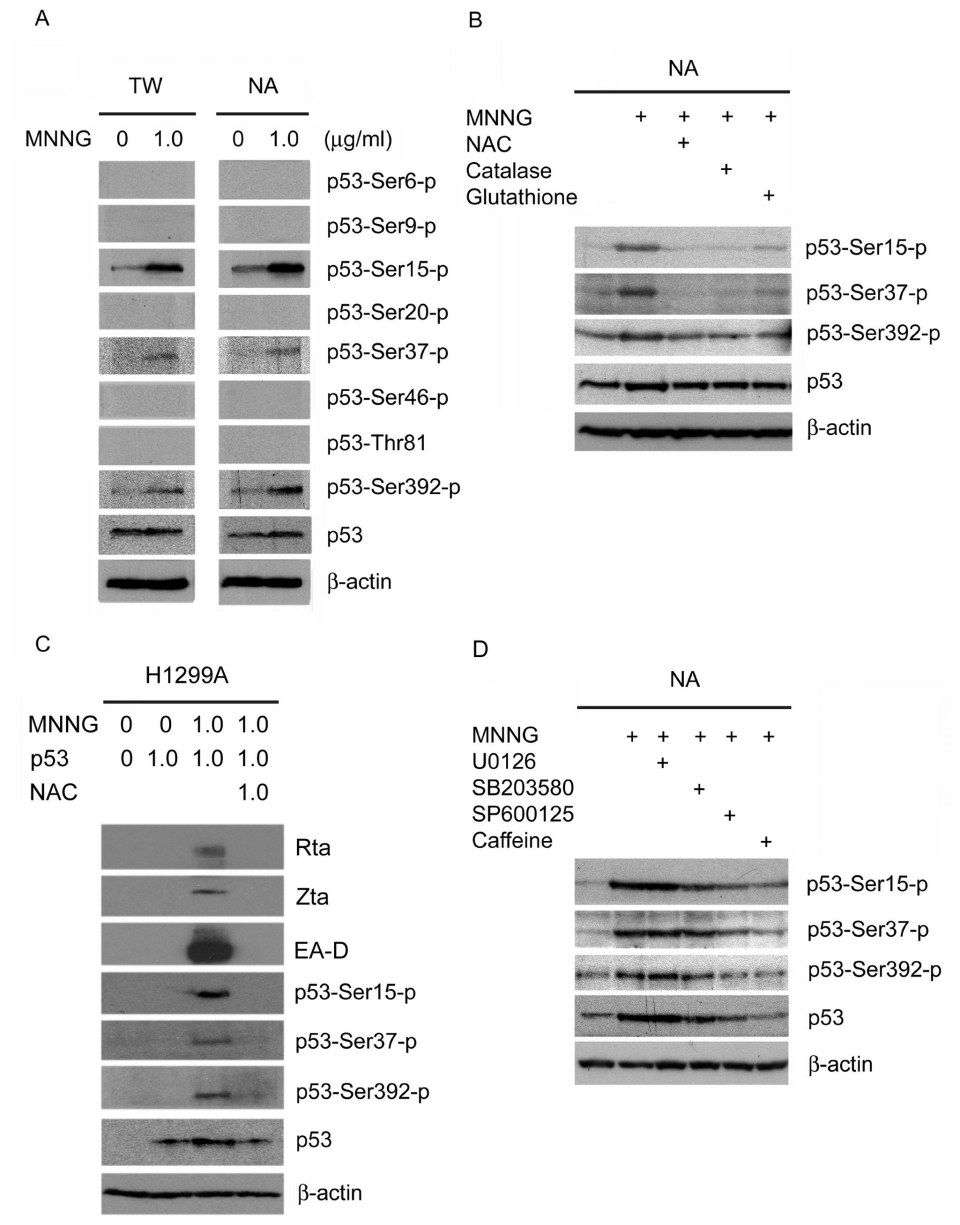
ATM, p38 MAPK and JNK are involved in the multiple phosphorylation of p53 by MNNG. (A) The phosphorylation profile of p53 protein following MNNG treatment. TW01 and NA cells were treated with MNNG for 24 h. Cell lysates were harvested and subjected to immunoblotting using antibodies against the indicated phosphorylation residues and total form of p53 protein. (B) ROS scavengers inhibited phosphorylation of p53 protein at Ser15, Ser37 and Ser392 and reduced accumulation of p53 protein after MNNG treatment in NA cells. After pretreatment with NAC (1mM), catalase (1000 unit/ml) or reduced glutathione (1mM) for 1 h and followed by MNNG (1μg/ml) treatment for another 24 h, NA cells were harvested and subjected to immunoblotting. (C) NAC inhibited EBV reactivation, phosphorylation of p53 protein at Ser15, Ser37 and Ser392, and reduced accumulation of p53 protein after MNNG treatment in H1299A cells. The cells were transiently transfected with a p53-expression plasmid for 24 h. After pretreatment with NAC (1mM) for 1 h and followed by MNNG (1μg/ml) treatment for another 72 h cell lysates were harvested and subjected to immunoblotting. (D) Inhibitors of p38 MAPK, JNK and ATM inhibited phosphorylation of p53 protein at Ser15, Ser37 and Ser392 and accumulation of the p53 protein. NA cells were pretreated with ERK inhibitor (U0126 10μM), p38 MAPK inhibitor (SB 203580 10μM), JNK inhibitor (SP600125 10μM) and ATM inhibitor (caffeine 1mM) for 1 h and then treated with MNNG (1μg/ml) for another 24 h. Cells were harvested and subjected to immunoblotting.

The functions of p53 are regulated by numerous post-translational modifications. From the above data, we hypothesized that post-translational modification of p53 protein by MNNG is critical for EBV reactivation. To test this hypothesis, the modification patterns of p53 protein were examined using antibodies against phospho-Ser6, -Ser9, -Ser15, -Ser20, -Ser37, -Ser46, -Thr81, and -Ser392 of p53 in MNNG-treated TW01 and NA cells. As shown in Figure 6A, phosphorylation of p53 protein was detected at Ser15, Ser37, and Ser392. Previously studies have shown that ROS induced phosphorylation of p53 protein is mediated via protein kinases, including ATM, ERK, p38 MAPK and JNK [50]. ROS scavengers were used to determine whether the phosphorylation events observed here were induced through ROS. As shown in Figure 6B, the phosphorylation of p53 protein at Ser15, Ser37, and Ser392, and the increase of p53 protein expression, were significantly attenuated by NAC (1mM), catalase (1000 unit/ml) and reduced glutathione (1mM) in NA cells. These data suggest MNNG induces the phosphorylation of p53 protein at these sites and the expression of p53 protein through ROS stimulation. In EBV-positive but p53-null H1299A cells, MNNG loses its ability to induce EBV reactivation suggesting p53 expression is critical for MNNG-induced EBV reactivation (Figure 5C). The results in Figure 5C also showed p53 over-expression alone without MNNG treatment is not sufficient to induce EBV reactivation. The EBV in H1299A cells can be reactivated only under restoration of p53 expression and MNNG treatment at the same time, suggesting p53 phosphorylation by MNNG may be important for EBV reactivation. In Figures 6B, the result showed MNNG induces the phosphorylation of p53 protein at Ser15, Ser37, and Ser392 through ROS. So we used NAC, a ROS scavenger to eliminate ROS, to address whether the p53 phosphorylation is induced by MNNG-mediated ROS production in the H1299A cell line. As shown in Figure 6C, NAC effectively inhibited MNNG induced p53 phosphorylation, suggesting p53 phosphorylation is a result of MNNG mediated ROS production. The EBV reactivation was also abolished by NAC while p53 phosphorylation was inhibited, which suggested that p53 phosphorylation induced by ROS may be necessary for MNNG mediated EBV reactivation. Furthermore, pretreatment of NA cells with SB203580 (10μM), SP600125 (10μM) and caffeine (1mM), but not U0126 (10μM), not only reduced the phosphorylation of p53 protein at Ser15, Ser37, and Ser392, but also blocked the increase of p53 protein expression (Figure 6D). These results indicate that the phosphorylation of p53 protein at these specific sites by MNNG is mediated by ATM, p38 MAPK and JNK, and post-translational modification of p53 protein may be required for p53 protein accumulation. 

### p53 binds to the Rta promoter

**Figure 7:**
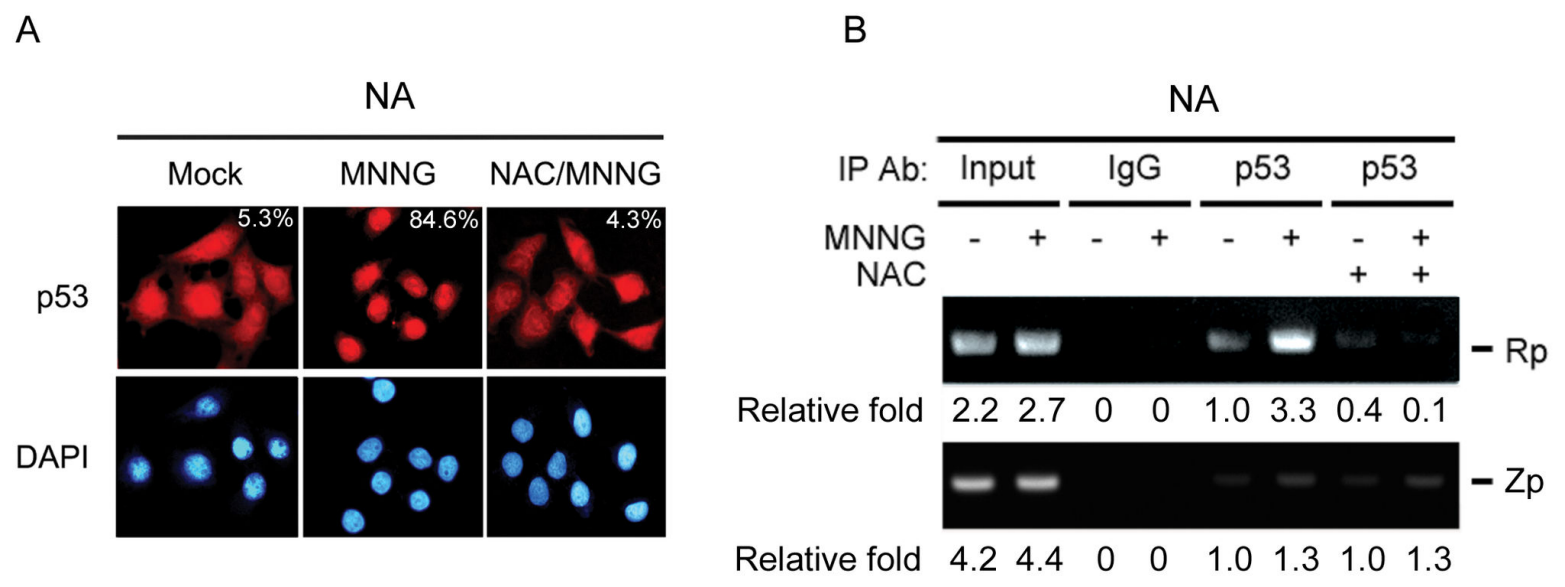
p53 binds to the Rta promoter. (A) MNNG induced the nuclear translocation of p53 protein. The localization of the p53 protein was determined using immunofluorescence analysis. NA cells were pretreated for 1 h with or without NAC (1mM) and then exposed to MNNG (1μg/ml) for an additional 24 h. Cells were analyzed using immunofluorescence staining for p53 localization. Representative photomicrographs were taken using a fluorescence microscope. Numbers indicates the percentages of cells with p53 translocation to the nucleus. (B) MNNG increased the binding of p53 to the Rta promoter. NA cells were mock treated or treated with MNNG (1μg/ml) for 24 h. Cell lysates were collected for ChIP assay using mouse monoclonal antibodies against p53 or mouse IgG as a control. PCR analysis of Rp (-442 to -2) and Zp (-221 to +12) were performed. Numbers indicates the relative fold compared to the control precipitated by p53 antibody.

Many genotoxic stresses stabilize the p53 protein and lead to its accumulation in the nucleus via initiating signaling pathways, and subsequently activate p53 protein as a transcription factor [53]. To determine whether MNNG induces the nuclear translocation of p53 protein, NA cells were treated with MNNG for 24 h and the localization of p53 protein was visualized by immunofluorescence. The localization of p53 protein was increased markedly in the nucleus after MNNG (1 μg/ml) treatment and this effect was highly attenuated by NAC pretreatment for 1 h (Figure 7A). The percentages of cells with p53 translocation to the nucleus were raised markedly by MNNG from 5.3% to 84.6%, but that was highly attenuated to 4.3% in the presence of NAC. This result suggests that MNNG induces the phosphorylation of p53 protein through ROS production and renders it to translocate and accumulate in the nucleus to function as a transcription factor. According to the result described above (Figure 5D), we assumed that p53 is involved in regulating Rp by binding to it. Therefore, the possibility of p53 binding to Rp or Zp was examined using a ChIP assay. As shown in Figure 7B, the amount of p53 on Rp significantly increased by MNNG at level about 3.3-fold higher than the solvent control. ROS scavenger, NAC, effectively inhibited the binding of p53 on Rp, suggesting p53 might regulate Rp activity by binding to it and the DNA-binding activity of p53 protein is regulated by ROS induced by MNNG. On the contrary, only slight 1.3-fold increase of p53 binding on Zp was detected by MNNG, and NAC treatment can not reverse this phenomenon. These results imply that p53 binding to Rp rather than to Zp induced by MNNG is more likely the mechanism for MNNG reactivation of EBV. 

According to sequence analysis, there is no typical consensus p53-response element (PRE) on the region of Rp we examined. However, it has been shown that p53 interacts with Sp1 to regulate Zp activity [52]. Because Sp1 binding sites are abundant on Rp, p53 protein may regulate Rp by interacting with Sp1 protein to bind indirectly to Sp1 binding sites. To investigate this possibility, a co-immunoprecipitation (Co-IP) assay was preformed in NA cells (Figure S1). p53 precipitated with Sp1 was increased about 1.4-fold compared to IgG on mock treatment and 1.8-fold on MNNG treatment. It also showed Sp1 precipitated with p53 was increased about 2.1-fold compared to IgG on mock treatment and 1.4-fold on MNNG treatment. Although this data is difficult to explain the role of MNNG in the interaction between p53 and Sp1, the interaction between p53 and Sp1 does exist. An EMSA assay was carried out to examine the possibility of interaction between the Sp1-binding element on Rp and transcription factor Sp1 or p53. The specific DNA-protein complexes were detected in the reactions (Figure S2). Furthermore, the DNA-protein complex on Sp1-binding element was abolished in the presence of antibody specific to Sp1 and p53. This result suggests that Sp1 and p53 may form a complex and bind on the Sp1-binding element of Rp. 

## Discussion

Previous epidemiological studies have shown that consumption of salted fish, preserved foods and smoking tobacco is associated with the development of NPC [7]. Volatile NOCs and their precursors are known to be present in preserved foods from NPC high risk areas and are considered to be an etiological factor for NPC [10,11]. However, the etiological mechanism has not yet been fully elucidated. A previous study has shown that Cantonese-style salted fish contain EBV reactivation-inducing substances [54], implying NOCs could induce viral replication. Recently, we showed that MNNG induces EBV reactivation [27] and enhances the genome instability and invasiveness, as well as the tumorigenicity, of NPC cells [28]. These studies reveal that EBV reactivation triggered by NOCs may play a crucial role in the course of NPC development. However, the molecular mechanism of the switch from latent EBV infection to lytic replication, induced by volatile NOCs, remains unclear. In contrast to preserved foods, frequent consumption of fresh fruits, fish, or vegetables, has been correlated with a lower risk of NPC [55-57]. Furthermore, intake of specific fruits or vegetables, including carrots, green leafy vegetables, fresh soybean products, oranges, tangerines, or dietary supplements of vitamin C or E has an inverse association with the risk of NPC [55,57]. The apparent protective effect of these foods may be attributed to their antioxidant properties, implying oxidative stress may play a critical role in the interplay between NOCs and EBV reactivation, contributing to carcinogenesis. Here, for the first time, we provide the evidence that MNNG induces ROS generation and causes ROS-mediated EBV reactivation via a p53-dependent mechanism. We also show that MNNG treatment leads to accumulation of p53 in the nucleus, increasing its binding to the Rp, and initiating viral replication via activation of the Rp. Notably, our results reveal the critical role of ROS in mediating the switch of EBV from the latent to the lytic phase by NOCs. 

We found that over 70% of NA cells were initiated into viral replication after MNNG treatment at a concentration of 1 μg/ml (Figure 1D, 1E). Low dose MNNG (0.1 μg/ml) did not induce detectable viral reactivation, but a significant increase of EBV reactivation was observed following long term and repeated treatment (Figure 1H). A previous study showed that total volatile N-nitrosamines in Chinese salted fish were 0.028 to 4.54 mg/kg [11]. A study of Thai cigarettes indicated that the yield of volatile nitrosamines in smoke was observed in ranges from 20.3 to 100.4 ng/per cigarette, while the tobacco-specific nitrosamines ranged from 88 to 1,580 ng/per cigarette [58]. Another study reported that bacon products contained about 0.5 μg/100 g of N-nitroso-dimethylamine, and after cooking the concentration seemed to increase [59], and sausages contained about 10 μg/100 g [60]. These reports suggest that the intake of NOCs may be sufficient to initiate EBV replication. Considering the physiological relevance that residents in NPC high risk areas may frequently consume preserved foods contained NOCs, EBV reactivation may often occur even if the exposure dosage on each occasion is low. 

Recent study showed that MNNG induces biphasic ROS production through NADPH oxidase and mitochondria [46]. It has been reported that H_2_O_2_ induces reactivation of Kaposi’s sarcoma-associated herpesvirus [61]. Here, we show that induction of oxidative stress by MNNG and H_2_O_2_ effectively induces EBV reactivation (Figure 3, 4). Similar data in a recent report indicated that H_2_O_2_ can induce EBV replication [49]. Moreover, several diseases, such as inflammatory bowel disease, rheumatoid arthritis and infectious mononucleosis, were found to be associated with EBV replication and meanwhile exhibit oxidative stress state [62-64]. Thus, oxidative stress resulting from physiological factors or chemical exposure could be an important factor that triggers EBV reactivation and consequently causes EBV-associated diseases. EBV infection and replication has been implicated in the generation of oxidative stress [35,36]. This may explain the accumulation of ROS in higher amounts in EBV-positive NA cells after reactivation than in EBV-negative TW01 cells following long term treatment with low dose MNNG (Figure 3F). Previous reports showed EBNA1 is responsible for the occurrence of oxidative stress in latent EBV infection [65]. However, the role of EBV replication or the expression of lytic viral antigens in the genesis of oxidative stress remains unclear. EBV Rta and Zta have been reported to alter mitochondrial membrane potential, which may imply an increase of ROS production [66]. The HCV core protein also has been shown to depolarize mitochondria to increase ROS production [67]. In addition, the HIV-1 gp120 and Tax were seen to induce oxidative stress [68]. The questions which viral lytic antigens are responsible and how they promote ROS generation will be an important focus of future studies. These results suggest that a positive feedback may be initiated as ROS production and EBV reactivation amplify each other and this may play a key role in the development and progression of EBV-associated diseases. Furthermore, the use of therapeutic drugs, such as chemotherapeutic and immunosuppressive drugs, known to induce oxidative stress, will need to be considered for the risk of concurrent diseases associated with EBV reactivation. Thus, it seems inhibition of ROS by combining antioxidants may be useful in the prevention or therapy of EBV reactivation-associated diseases. 

H_2_O_2_ has been shown to induce Zta transcription [69]. A previous study showed the KSHV R homologue (RTA) can be activated by H_2_O_2_, leading to RTA-mediated viral reactivation [61]. In addition, the switch from latent to lytic EBV infection is known to be regulated by expression of either the Rta and/or Zta immediately early proteins [70]. Rta and Zta activate one another’s promoters and cooperatively activate the early lytic viral promoters [45,71]. Current models have favored that Zta plays the dominant role in reactivation of EBV through activating transcription by binding to Zta response elements (ZRE) on promoters of Rp and Zp [72]. Expression of Zta efficiently initiates the entire lytic cascade in B lymphocytes [70,73]. It has been reported that the transcription of a class of viral lytic genes depends on Zta, and EBV is not able to complete lytic cycle without Zta expression [45,74]. Rta drives EBV gene expression by directly binding to responsive promoters that contain Rta response elements (RRE) or by an indirect mechanism [72]. Although Rta synergizes with Zta in the activation of many viral genes, Rta remains capable to transactivate certain downstream viral genes. Some early and late genes, such as BaRF1, BMLF1, and BLRF2, are activated by Rta itself in the absence of Zta [75]. A class of lytic cycle genes, such as BMRF1 and BHRF1, are activated in synergy by Rta and Zta (S186A) mutant, whose transactivation function is manifested only in Rta coexpression [75]. Previous studies have shown that expression of Rta activates lytic EBV replication in B lymphocytes, and can activate the transcription of BRLF1 to autoregulate its own expression [71]. In those cells, Rta leads to activation of Zp, expression of Zta, and consequently stimulation of all the lytic gene expression. In this study, we show that the amount of Rta mRNA was increased about 2.2-fold by MNNG treatment (1 μg/ml) for 24h, while the amount of Zta mRNA was not significantly increased (Figure 1F). However, the amount of Zta mRNA was significantly increased to 16.6-fold by MNNG treatment (1 μg/ml) for 72h. The reporter assays by MNNG treatment (1 μg/ml) for 24h also show the remarkable response of Rp activity than that of Zp on MNNG treatment (Figure 1G, 3E). The results suggest that MNNG initiates EBV reactivation may mainly through induction of Rp activation to induce expression of Rta. It is difficult to examine whether Rta expression alone can reactivate EBV under MNNG treatment, as Zta is expressed upon induction of the lytic cycle. In light of previous work that implicating Rta by itself has less ability than Zta to activate most lytic genes, we suggest a cooperative model for EBV entry into the lytic cycle under MNNG treatment. Expression of Rta triggers expression of the Zta, and together act in synergy to activate the viral lytic cycle. Furthermore, p53 is crucial for MNNG to induce the activation of the Rp. We also showed that knockdown of endogenous p53 expression diminishes the ability of MNNG to induce EBV reactivation (Figure 5A, B) while loses its effect on the activation of the Rp (Figure 5D). Furthermore, we showed that MNNG cannot induce EBV reactivation in H1299A cells unless the expression of p53 protein is restored (Figure 5C). Thus, our results suggest that MNNG-induced EBV reactivation may be executed by the activation of Rp by p53. 

Many signaling pathways and transcription factors appear to be regulated by ROS [76]. Among them, MAPKs, PI3K, PKC, ATM, AP-1, Sp-1, p53, and NFκB have been reported to be involved in the activation of EBV replication [48,51,52,77,78]. In this study, only certain signaling pathways involving both ROS and EBV replication were tested. Our results show that the ability of MNNG to induce EBV reactivation is mediated through at least the ATM, p38 MAPK, and JNK pathways activated by ROS (Figure 2). Earlier studies have established that p53 is activated by ROS and plays an important role in response to ROS functions [50]. In addition, it is important to note that p53 participates in EBV replication [51,52]. Here, we show that ATM, p38 MAPK and JNK activated by MNNG-induced ROS are involved in the multiple phosphorylation of p53 at Ser-15, Ser37, and Ser-392 (Figure 6). Moreover, p53 is known to be phosphorylated and consequently stabilized and activated by multiple signaling pathways, including ATM and MAPKs [79,80]. We found that MNNG treatment significantly induced phosphorylation and stabilization of p53, and these events were inhibited by ATM, p38 MAPK and JNK inhibitors (Figure 6D). This finding is consistent with previous studies suggesting that post-translational modification of p53 by these kinases might increase the stability of p53 protein. The p38 MAPK and JNK phosphorylated transcription factor ATF2 has been reported to activate the Zp [77], implying these kinases might similarly affect the Rp through modification of other transcription factors, such as p53. Thus, we hypothesized that the major role of ROS during initiation of EBV reactivation might be in enhancing the p53 transcriptional function via activation of ATM, p38 MAPK and JNK. Our data show that the treatment of cells with MNNG (1μg/ml) through ROS production resulted in both p53 activation and EBV reactivation (Figure 5, 6). Our data also indicate that p53 activation by post-translational modification is required for MNNG-induced EBV reactivation (Figure 5, 6). Furthermore, our data show that a lower level of p53 activation induced a lower level of EBV reactivation, whereas a higher level of p53 activation induced a higher level of EBV reactivation (Figure 5C), suggesting the activation of p53 seems to be critical for regulating EBV reactivation. 

N-nitroso compounds (NOCs) also have long been known as alkylating agents, which are capable of reacting with DNA and generate alkylating DNA adducts by formation of reactive diazonium ion species[3]. O-6-alkylguanin has been identified as the main predominant mutagenic and cytotoxic lesion because of the mispairing properties, which causes point mutation and chromosomal aberration [3,81]. The lesion caused by alkylating agents not only appears to be involved in genotoxic stress but also provides the primary signal activating specific molecules and signaling pathway to trigger DNA damage response[82]. On the other hand, nitrosamines have been reported to increase the formation of radical intermediates including HO·, NO·, alpha-hydroxynitrosamines and N-methylformaldimine in the metabolic process[83]. It also has been shown that NOCs caused formation of ROS and carbon-centered radicals, which play an important role in deregulation of gene expression patterns of apoptosis, cell cycle blockage, DNA repair, and oxidative stress [84,85]. p53 has long been recognized as center of the sensor and responder in response to DNA damage [86]. Previous reports have shown that alkylating agents such as MNNG and MMS, induce an increase in phosphorylation and protein level of p53 through functional protein complexes of mismatch repair in DNA damage response [82,87]. These data suggest that DNA lesions caused by both of alkylating and oxidative DNA damage could be the primary signal to trigger DNA damage response and then induce p53 activation. Previous study have also shown that DNA damage response could induce EBV reactivation [49], thus DNA damage response could be part of the mechanism involving MNNG stabilizes and activates p53 to induce EBV reactivation. In general the stability of p53 is under strict control by its negative regulator MDM2 and activated by a post-translation leading to stabilization [80]. It has been reported NOCs act as mitomycin C (MMC) and MMS in stabilization and accumulation of p53 through downregulation of MDM2 mRNA and protein [88]. Furthermore, recent reports showed p63 and p73, two related p53 families, in part regulated for p53 recruiting and function, and interacted with p53 in response to DNA damage [89,90]. Interestingly, p63 and p73 can also interact with MDM2 and MDM4, but MDM2 and MDM4 do not cause p63 or p73 degradation [91]. This result may imply p63 and p73 regulating p53 stability in part through interaction of MDM2 and MDM4. These reports suggest that the post-translational modification of p53 may not be the only mechanism to regulation of p53 stability and activity in the cells treated with NOCs. Although we demonstrated MNNG induce phosphorylation and stabilization of p53 through ROS in this study, the possibility of other factors such as the p63/p73 isoforms involved in the activation of p53 by MNNG to induce EBV reactivation should also be considered. 

p53 is a potent activator of cellular transcription via binding to the promoter regions of its target genes [80]. It has been shown that p53 participates in lytic EBV reactivation by forming a complex with Sp1 that binds to, and activates, the Zp [52]. In this study, we found that MNNG, through ROS, promotes p53 translocation into the nucleus (Figure 7A) and increases the ability of p53 to bind to the Rp (Figure 7B). Promoter recognition by p53 is determined by the presence of p53-response elements (PREs) with the consensus sequence of decamers 5’-(PuPuPuC(A/T)(T/A)GPyPyPy)n-3’[92]. However, the nucleotide sequences of decamers usually violate the typically consensus sequence in PREs of p53-regulated genes (e.g. c-Ha-ras, mck, pig3, fas/apo1, tgf-α) [93]. Although there are no typically consensus PREs on the region of Rp we examined, p53 might possibly bind onto Rp via a non-consensus binding sequence. Thus, further study is required to determine whether p53 can bind directly to the Rta promoter. On the other hand, p53 was seen to form a complex with Sp1 (Figure S1) and we also found that both Sp1 and p53 can bind on Rp in EMSA assay (Figure S2). Therefore, these results suggest that p53 might bind to the Rp directly or at least by forming a complex with Sp1, and then activate the Rp during MNNG initiation of EBV reactivation through its DNA binding and transactivational function. Thus, p53 plays an important regulatory role in promoting the switch between latent and lytic EBV infection in epithelial cells when activated by MNNG.

**Figure 8:**
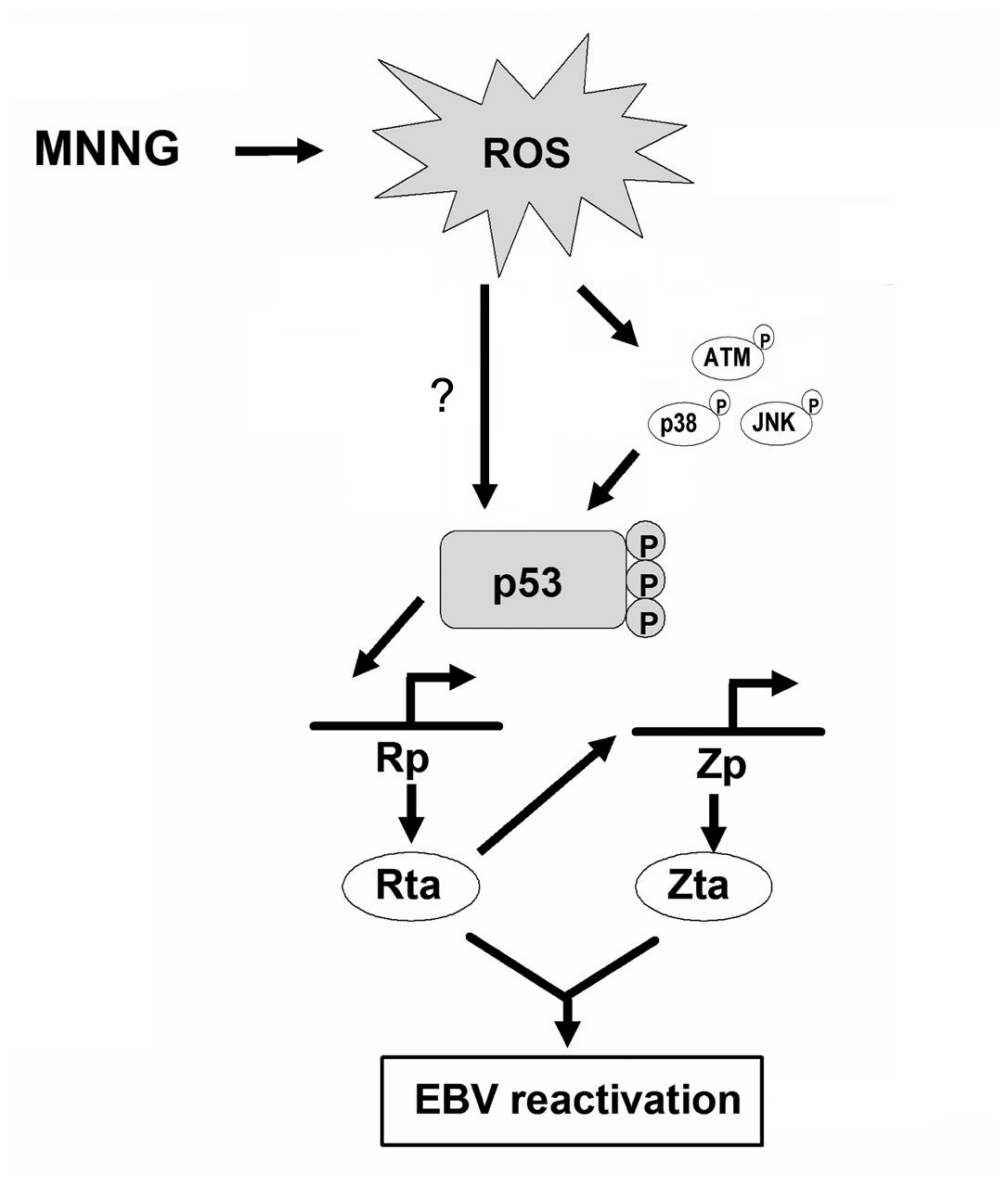
Model of the regulation of EBV reactivation by MNNG. In this study, we demonstrated that MNNG induces ROS production. Subsequently, ROS induces the activation of ATM, p38 MAPK, and JNK and then leads to p53 activation. Activated p53 binds to and subsequently activates the promoter of the EBV immediate early protein Rta (Rp). Induction of Rp activity leads to Rta expression, which reciprocally induced Zta expression. The synergistic effect of Rta and Zta then induce the expression of all the lytic proteins. In addition, ROS may also induce the p53 activation through additional pathways other than ATM/p38 MAPK/JNK, and these mechanisms should also be considered.

Together, the results presented here suggest a model (Figure 8) in which MNNG induces ROS generation to activate ATM, p38 MAPK, and JNK signaling, leading to phosphorylation and activation of p53. On the other hand, the possibility of others factors activated by ROS may involve in this mechanism should also be considered. The activated p53 translocates and accumulates in the nucleus, which then binds to and activates the EBV Rp. The expression of Rta subsequently leads to activation of Zta expression, and the synergistic effect of Rta and Zta then induce the expression of all the lytic proteins. 

## Supporting Information

Figure S1 **p53 interacted with Sp1.** NA cells were mock treated or treated with MNNG (1μg/ml) for 24 h and then harvested for Co-IP assay using mouse monoclonal antibodies against p53, Sp1, or mouse IgG as a control. Cells were trypsinized and lysed with RIPA buffer (50 mM HEPES, pH 7.5, 250 mM NaCl, 5 mM EDTA, 1 mM dithiothreitol , 0.1% NP-40, 1 mM NaF, 0.1 mM Na3VO4), containing complete protease inhibitor cocktail (Roche, Nutley, NJ). Cell lysates were pre-cleared with 100 μl protein G-Sepharose beads for 2 h and incubated with antibodies specific for Sp1 or p53 (4 μg each) at 4°C overnight on a rotating rocker. Immunocomplexes were collected using 200 μl of protein G-Sepharose beads at 4°C for another 2 h. The immunocomplexes bound to Sepharose beads were washed extensively with ice-cold RIPA buffer. The precipitates were boiled in Laemmli sample buffer and resolved by SDS-polyacrylamide gel electrophoresis. Immunocomplexes were revealed by immunoblotting.
(TIF)

Figure S2 **Sp1 and p53 may form a complex and bind on the Sp1-binding element of Rp.** Oligonucleotides of Sp1-binding element on the -58 to -35 region of Rp (5’-CGATT GTCCC GCCCA TGCCA ATGG-3’) were synthesized and labeled with biotin (Purigo Biotech, Inc.). The binding reaction was performed in a 20 μl reaction mixtures containing 8 μg of nuclear extracts from NA cells, 1 μM Biotin-labeled probes, 20 mM Tris-HCl, 50 mM KCl, 5 mM MgCl2, 0.5 mM EDTA, 1 mM dithiothreitol, 10% glycerol, and 2 μg of poly (dI-dC). Two μg of anti-Sp1#1 (SC-59; Santa Cruz, CA), anti-Sp1#2 (SC-420X; Santa Cruz), anti-p53 (SC-126; Santa Cruz) antibodies or mouse IgG as a control were respectively added to the reactions for antibody supershift in the electrophoretic mobility shift assay (EMSA). All reactions were incubated for 30 min at room temperature and then electrophoresed at 110V in 6% native polyacrylamide gels with Tris-borate buffer (90 mM Tris, 90 mM boric acid, 2 mM EDTA). The DNA-protein complexes were then electrophoretically transferred to Hybond-N membrane (Amersham). The biotin-labled DNA was detected by chemiluminescence using the LightShift Chemiluminescence EMSA kit (Pierce) according to the manufacturer’s recommendations and exposed to X-ray film.
(TIF)
