# Supplementary figures and images for: Reactive Oxygen Species Mediate Epstein-Barr Virus Reactivation by N-Methyl-N’-Nitro-N-Nitrosoguanidine
Source: PLoS One. 2013 Dec 20;8(12):e84919. doi: 10.1371/journal.pone.0084919 (PMC3869928; doi:10.1371/journal.pone.0084919)

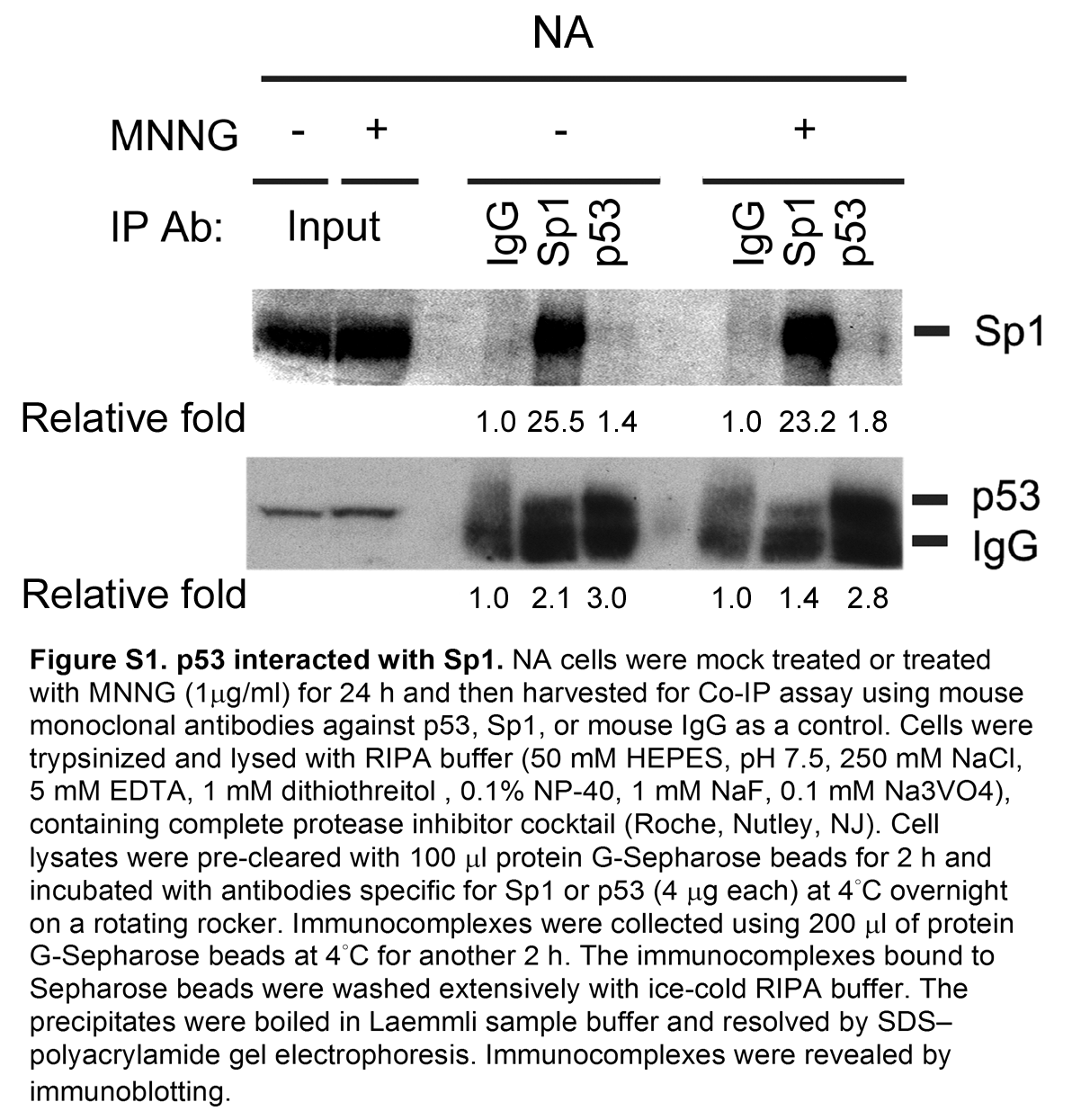

Supplement: Figure S1 — p53 interacted with Sp1. NA cells were mock treated or treated with MNNG (1μg/ml) for 24 h and then harvested for Co-IP assay using mouse monoclonal antibodies against p53, Sp1, or mouse IgG as a control. Cells were trypsinized and lysed with RIPA buffer (50 mM HEPES, pH 7.5, 250 mM NaCl, 5 mM EDTA, 1 mM dithiothreitol , 0.1% NP-40, 1 mM NaF, 0.1 mM Na3VO4), containing complete protease inhibitor cocktail (Roche, Nutley, NJ). Cell lysates were pre-cleared with 100 μl protein G-Sepharose beads for 2 h and incubated with antibodies specific for Sp1 or p53 (4 μg each) at 4°C overnight on a rotating rocker. Immunocomplexes were collected using 200 μl of protein G-Sepharose beads at 4°C for another 2 h. The immunocomplexes bound to Sepharose beads were washed extensively with ice-cold RIPA buffer. The precipitates were boiled in Laemmli sample buffer and resolved by SDS-polyacrylamide gel electrophoresis. Immunocomplexes were revealed by immunoblotting. (TIF) [file pone.0084919.s001.tif]

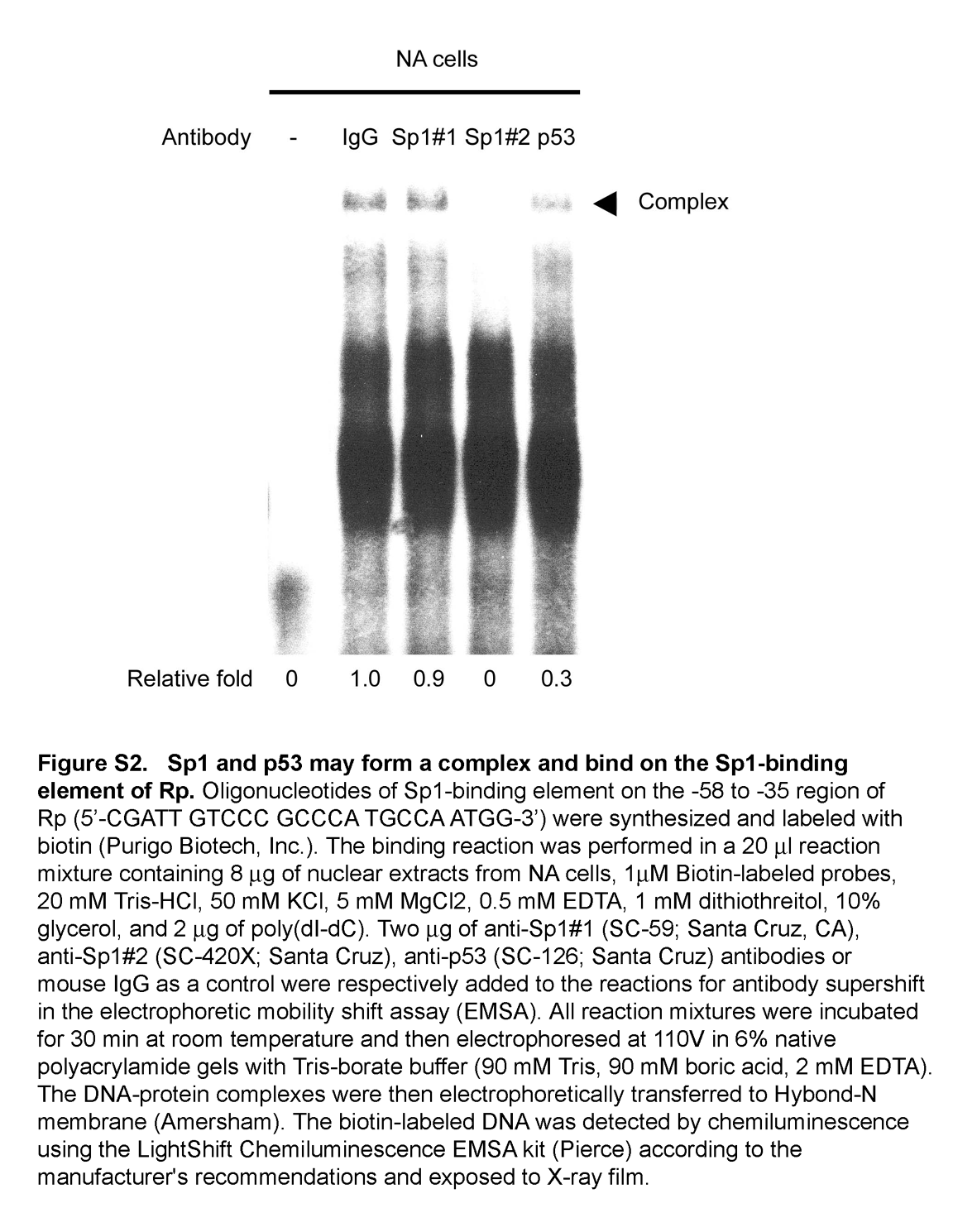

Supplement: Figure S2 — Sp1 and p53 may form a complex and bind on the Sp1-binding element of Rp. Oligonucleotides of Sp1-binding element on the -58 to -35 region of Rp (5’-CGATT GTCCC GCCCA TGCCA ATGG-3’) were synthesized and labeled with biotin (Purigo Biotech, Inc.). The binding reaction was performed in a 20 μl reaction mixtures containing 8 μg of nuclear extracts from NA cells, 1 μM Biotin-labeled probes, 20 mM Tris-HCl, 50 mM KCl, 5 mM MgCl2, 0.5 mM EDTA, 1 mM dithiothreitol, 10% glycerol, and 2 μg of poly (dI-dC). Two μg of anti-Sp1#1 (SC-59; Santa Cruz, CA), anti-Sp1#2 (SC-420X; Santa Cruz), anti-p53 (SC-126; Santa Cruz) antibodies or mouse IgG as a control were respectively added to the reactions for antibody supershift in the electrophoretic mobility shift assay (EMSA). All reactions were incubated for 30 min at room temperature and then electrophoresed at 110V in 6% native polyacrylamide gels with Tris-borate buffer (90 mM Tris, 90 mM boric acid, 2 mM EDTA). The DNA-protein complexes were then electrophoretically transferred to Hybond-N membrane (Amersham). The biotin-labled DNA was detected by chemiluminescence using the LightShift Chemiluminescence EMSA kit (Pierce) according to the manufacturer’s recommendations and exposed to X-ray film. (TIF) [file pone.0084919.s002.tif]
